# Effect of the Immobilization Strategy on the Efficiency and Recyclability of the Versatile Lipase from *Ophiostoma piceae*

**DOI:** 10.3390/molecules24071313

**Published:** 2019-04-03

**Authors:** María Molina-Gutiérrez, Neumara L. S. Hakalin, Leonor Rodríguez-Sánchez, Lorena Alcaraz, Félix A. López, María Jesús Martínez, Alicia Prieto

**Affiliations:** 1 Centro de Investigaciones Biológicas (CSIC), C/Ramiro de Maeztu, 9, 28040 Madrid, Spain; mariamg@cib.csic.es (M.M.-G.); nhakalin@cib.csic.es (N.L.S.H.); leonor@cib.csic.es (L.R.-S.); 2 Centro Nacional de Investigaciones Metalúrgicas (CSIC), C/Gregorio del Amo, 8, 28040 Madrid, Spain; alcaraz@cenim.csic.es (L.A.); f.lopez@csic.es (F.A.L.)

**Keywords:** short-chain fatty acids esters, biocatalysis, recycling, green chemistry, fragrances, flavours

## Abstract

The recombinant lipase from *Ophiostoma piceae* OPEr has demonstrated to have catalytic properties superior to those of many commercial enzymes. Enzymatic crudes with OPEr were immobilized onto magnetite nanoparticles by hydrophobicity (SiMAG-Octyl) and by two procedures that involve covalent attachment of the protein (mCLEAs and AMNP-GA), giving three nanobiocatalysts with different specific activity in hydrolysis of *p*-nitrophenyl butyrate (*p*NPB) and good storage stability at 4 °C over a period of 4 months. Free OPEr and the different nanobiocatalysts were compared for the synthesis of butyl esters of volatile fatty acids C4 to C7 in reactions containing the same lipase activity. The esterification yields and the reaction rates obtained with AMNP-GA-OPEr were in general higher or similar to those observed for the free enzyme, the mCLEAs-OPEr, and the non-covalent preparation SiMAG-Octyl-OPEr. The time course of the esterification of the acids C4 to C6 catalyzed by AMNP-GA-OPEr was comparable. The synthesis of the C7 ester was slower but very efficient, admitting concentrations of heptanoic acid up to 1 M. The best 1-butanol: acid molar ratio was 2:1 for all the acids tested. Depending on the substrate, this covalent preparation of OPEr maintained 80–96% activity over 7 cycles, revealing its excellent properties, easy recovery and recycling, and its potential to catalyze the green synthesis of chemicals of industrial interest.

## 1. Introduction

Lipases (EC 3.1.1.3) are pivotal catalysts for organic synthesis. Due to their catalytic versatility, they are among the most important enzymes for industrial applications [1,2]. In their natural environment they catalyze the hydrolysis of lipids in aqueous media, but under low water-content conditions they can promote the synthesis of esters by esterification, transesterification, alcoholysis or acidolysis, among other reactions. 

However, several facts limit the use of lipases for some applications at an industrial scale. Their high cost and the possibility of enzyme inactivation by the acids and alcohols used as substrates are some of the most outstanding issues. For example, short-chain carboxylic acids tend to partition to the microenvironment around the lipase decreasing the local pH, which can affect catalytic activity [3], and alcohols, which are competitive inhibitors of lipases, can irreversibly inactivate lipases [4]. In this context, immobilization can be an excellent choice to improve the catalyst’s stability and recovery decreasing the production costs [1]. The most common techniques used for protein immobilization involve their simple entrapment (encapsulation), their attachment to a support, or the preparation of cross-linked enzyme aggregates (CLEAs) with or without a carrier [5,6,7,8]. Except in the first case, the protein establishes an interaction with the carrier, either by physical adsorption through hydrophobic or van der Waals interactions, by ionic binding or by covalent attachment or crosslinking. Materials that range from biopolymers to inorganic compounds can be used as carriers and among them nanomaterials offer several advantages. For example, they have larger specific surface area and less mass transfer limitations than other materials [9]. In particular, magnetic nanoparticles (MNPs) have unique additional properties such as their superparamagnetic behavior and their easy separation under an external magnetic field. To protect the magnetic core and improve enzyme activity, the MNPs require coatings on their surface [6,9] that can be further modified by specific functional groups (epoxy, amino, carboxylate, thiol, alkyl, etc.).

Non-covalent methods that rely in physical adsorption are cheap and simple, as the process is performed by direct contact of enzyme and carrier at mild temperature and do not require any chemical compounds. Since lipases are very hydrophobic proteins, they can be easily adsorbed on hydrophobic carriers functionalized with alkyl chains [10].

However, for many applications, covalent attachment is preferred because it prevents from leaching of the enzyme [5]. When the support is aminated (AMNP), bifunctional reagents such as glutaraldehyde (GA), can be used for activation. In the appropriate conditions, the free aldehyde group from the linker forms covalent imine bonds with reactive amino groups of the biomolecule [11]. GA can also be used for covalent immobilization as magnetic cross-linked enzyme aggregates (mCLEAS), which involves the precipitation of soluble proteins in the presence of MNPs, and the subsequent cross-linking of the mixture with GA [7,12,13,14,15]. 

Thus, the properties of the final catalyst depend on the way the protein is linked to the carrier, or in other words, on the type of carrier, the functional group in its surface and the immobilization conditions [10,16,17]. In this work, we have assayed the immobilization on MNPs of the non-commercial lipase OPEr, a recombinant form of the enzyme naturally secreted by the fungus *Ophiostoma piceae* (OPE) heterologously produced in *Pichia pastoris*. This enzyme is a versatile lipase from the *Candida rugosa*-like family, with high activity on triglycerides and sterol esters [18]. OPE, and specially OPEr, have been tested in hydrolysis and synthesis reactions in their soluble form, revealing that they are versatile enzymes with catalytic efficiencies superior to those reported for other lipases [19,20,21,22,23,24]. In view of its biotechnological potential, we tackled the immobilization of OPEr according to three strategies in order to compare their activity with that of the soluble enzyme. One of the approaches was non-covalent immobilization by hydrophobic interaction, and the other two procedures involved the use of GA-activated AMNPs for covalent attachment of the lipase either directly or by forming mCLEAs. 

The magnetic nanobiocatalysts were tested in the synthesis of esters of short chain volatile fatty acids (VFA), because these compounds have potential biotechnological interest and offered a simple model to evaluate the influence of the chain-length of the substrate in the enzymatic activity. These esters, that contribute to the natural aroma and taste of fruits and vegetables, are profusely used as additives in the pharmacy, cosmetics, and food industries [25,26] and can be extracted in very low concentration from natural sources [27,28]. However, for industrial purposes, they are generally obtained by chemical transformation at high temperatures with non-selective catalysts. Under these conditions, unwanted secondary products are generated [2,26], and the esters cannot be labelled as natural products [25,28,29]. Now, the regulations promoting the production of natural ingredients, summed to the consumers’ preference for natural foodstuffs have fostered the importance of bio-based chemicals. The synthesis of esters by biocatalysis overcomes the above issues, as the reactions are specific, selective, clean, and developed under mild conditions. Hence, the esters enzymatically produced comply with the European and American regulations for natural compounds [25,26] being the expected annual growth rate of their global market of around 6.4% for 2016–2021 (BBC, 2016). Lipases of the fungi *C. rugosa*, *Candida antarctica, Rhizopus oryzae*, *Thermomyces lanuginosus*, or *Rhizomucor miehei* are among the most frequently reported biocatalysts for the synthesis of aroma esters, generally by direct esterification of VFA and alcohols [1,2,26,30]. These are well-known commercial catalysts used in a wide array of reactions. Apart from lipases, other enzymes from the α/β hydrolase family have demonstrated their ability to produce these aliphatic esters. For example, esterifications catalyzed by cutinases have shown to be very efficient with substrates with 4–7 carbon atoms, and several reports deal with their use to produce of short-chain esters [31,32,33,34,35].

This study gathers the results of three approaches for immobilization of the lipase OPEr on MNPs. The simplest method was based on the non-covalent hydrophobic interaction of the lipase with a commercial magnetic carrier harboring hydrophobic octyl groups in the surface. On the other hand, the two methodologies applied for covalent immobilization of OPEr used GA-activated AMNPs as carrier. To form covalent mCLEAs, the protein solution is allowed to interact with GA-activated AMNPs for a short period before adding the precipitant and the crosslinker. Finally, the third protocol involved the formation of a covalent imine between GA-activated AMNPs and amino groups of the protein. The three magnetic preparations with immobilized OPEr were assayed as catalysts of the synthesis of the butyl esters of volatile fatty acids of different chain-length. The best nanobiocatalyst was selected to study the influence of several parameters in the esterification yields and the operational stability of the preparation with the C4-C7 fatty acid substrates.

## 2. Results 

### 2.1. Immobilization of Enzyme Crudes Containing the Lipase OPEr

The versatile lipase OPEr (the recombinant lipase from *Ophiostoma piceae*) was immobilized on the surface of magnetic nanoparticles by three procedures. The nanoparticles used for covalent attachment were prepared by treatment of commercial nude magnetite with APTS to obtain silanized and amino-functionalized nanoparticles (AMNPs) that were further activated with glutaraldehyde before protein attachment (AMNP-GA-OPEr). The same activated AMNPs were used to prepare mCLEAs-OPEr, adding the protein, ammonium sulfate as precipitant agent, and glutaraldehyde as cross-linker. Finally, non-covalent immobilization involved the hydrophobic interaction of OPEr with a commercial magnetic carrier functionalized with octyl groups (SiMAG-Octyl). This procedure yielded a catalyst with virtually pure lipase (SiMAG-Octyl-OPEr), since OPEr is not only the major protein in the crudes, but also the most hydrophobic. Table 1 presents the data on the total protein and lipase activity offered for each immobilization strategy, as well as the immobilization yields and the specific activity of the final nanobiocatalysts. According to the values of residual activity measured in the supernatants after immobilization, nearly all the lipase offered was immobilized by hydrophobic interaction on SiMAG-Octyl (99%). Concerning the two covalent enzyme preparations, 97% of the offered OPEr was immobilized as mCLEAs while 53% of the lipase was anchored as AMNP-GA-OPEr. The activity of the nanobiocatalysts was tested in independent reactions containing the same weight of each biocatalyst and *p*NPB as substrate. 

The specific activities determined for SiMAG-Octyl-OPEr and AMNP-GA-OPEr were comparable (430–440 mU/mg, respectively) in spite of the higher immobilization yields observed for the first carrier. However, the specific activity of the mCLEAs-OPEr on this substrate was considerably higher.

The characterization of the nanoparticles and biocatalysts is presented in Section 2.6.

### 2.2. Activity of the Nanobiocatalysts in Synthesis of Butyl Esters of Volatile Fatty Acids

In order to compare the activity of the free enzyme and the three immobilized preparations we assayed the synthesis of the butyl esters of a battery of linear volatile fatty acids from four (C4) to seven (C7) carbon atoms. These experiments will serve to find differences in the reaction rates and efficiencies of the catalysts, and to select one of the nanobiocatalysts to conduct further assays and evaluate its recyclability. 

**Figure 1:**
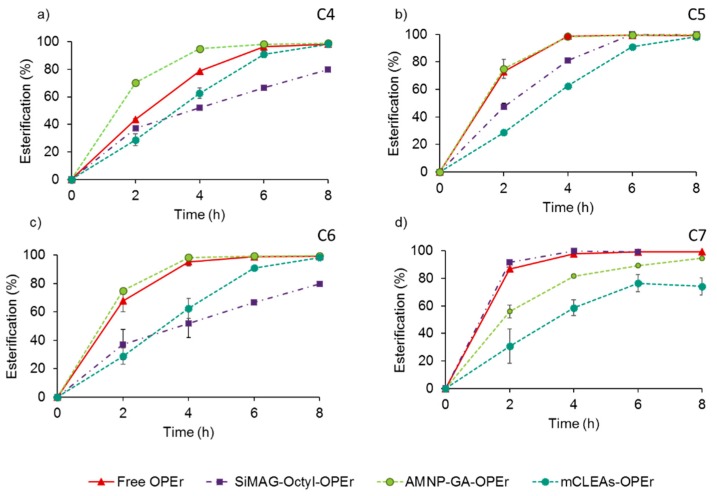
Comparison of the time-course of the esterification reactions, catalyzed by free OPEr and three preparations of the same enzyme immobilized on magnetic nanoparticles. The substrates were 1-butanol and 100 mM of the acids in isooctane: (**a**) butanoic (C4); (**b**) pentanoic (C5); (**c**) hexanoic (C6); and (**d**) heptanoic (C7) acid. Reactions (final volume 500 µL) were performed at 100 rpm and 25 °C, with 11 U of catalyst and a 2:1 molar ratio 1-butanol: VFA (volatile fatty acids).

The plots in Figure 1 illustrate the time course of 8-h reactions catalyzed by crudes with free OPEr and the nanobiocatalysts, using as substrates straight-chain volatile fatty acids (VFA) from C4 to C7 and the linear alcohol 1-butanol, with isooctane as co-solvent. The results evidenced the effectiveness of OPEr in all the forms tested, as all catalysts were able to esterify each substrate in the standard conditions established (25 °C, 100 rpm, 100 mM VFA, molar ratio 1-butanol:VFA 2:1, 11 U of catalyst). In general, SiMAG-Octyl-OPEr showed lower efficiency than the other catalysts, which esterified more than 80% of each limiting substrate in 6 h. However, in all instances, the synthesis catalyzed by mCLEAs-OPEr proceeded more slowly than with AMNP-GA-OPEr, and even with SiMAG-Octyl-OPEr for the C5 and C7 compounds. The trend observed for esterification of the acids C4-C6 changed for heptanoic acid, since the non-covalent nanobiocatalyst and the free enzyme achieved 90% conversion of this substrate in the two first hours. 

These results show that, for three out of the four butyl esters assayed, the synthesis catalyzed by the covalent AMNP-GA-OPEr equaled or surpassed the efficiency of the free enzyme, the non-covalent nanobiocatalyst and the mCLEAs. 

### 2.3. Storage Stability

**Figure 2:**
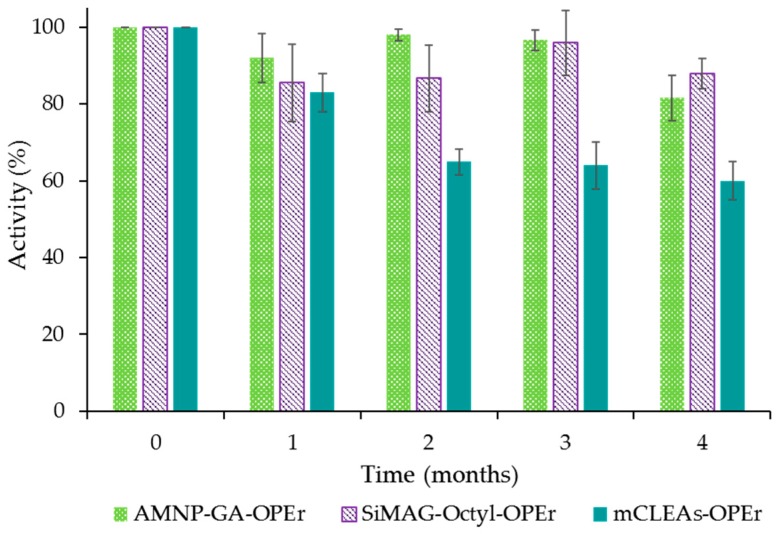
Activity of the three biocatalysts with immobilized OPEr, during storage for four months at 4 °C in Tris-HCl 20 mM pH 7, without stabilizers.

The stability of the immobilized catalysts across a prolonged period is an important feature for their potential application. Thus, we assessed the activity of the immobilized forms of OPEr presented in this study over four-months storage at 4 °C in Tris-HCl 20 mM pH 7, without stabilizers’ addition. The data in Figure 2 display the results from the aliquots taken monthly. The mCLEAs lost more than 40% of the initial activity after the first month. The other nanobiocatalysts maintained more than 80% of the activity measured just upon immobilization over four months in the conditions assayed. Since enzyme leakage was discarded, as no activity or proteins were detected in the storage buffer, the activity losses could be due to OPEr inactivation. 

Due to its improved efficiency on most of the substrates tested and because of its good stability after long-term storage, the covalent preparation AMNP-GA-OPEr was selected for the following experiments.

### 2.4. Effect of Several Reaction Parameters in the Synthesis of Butyl Esters of Volatile Fatty Acids Catalyzed by AMNP-GA-OPEr

#### 2.4.1. Molar Ratio of Substrates

**Figure 3:**
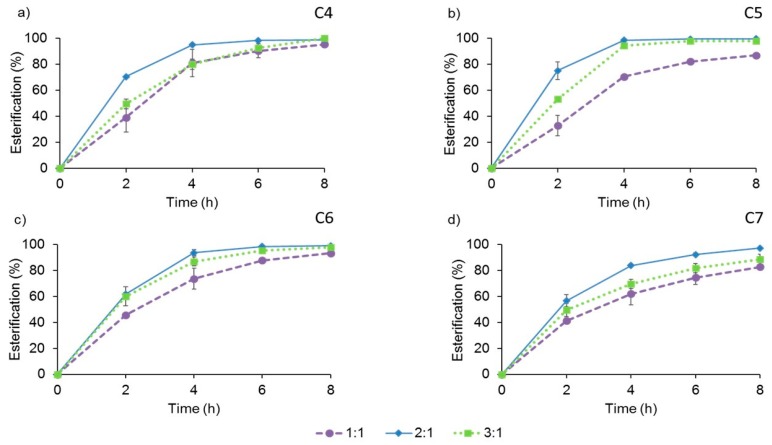
Effect of the molar ratio of 1-butanol and each VFA on the time-course of the esterification catalyzed by AMNP-GA-OPEr: (**a**) butanoic (C4); (**b**) pentanoic (C5); (**c**) hexanoic (C6); and (**d**) heptanoic (C7) acid. Reactions were performed in isooctane, at 100 rpm and 25 °C, with 11 U of catalyst and 100 mM of each VFA.

In addition to the 2:1 proportion, established for the standard reactions, we also assayed lower (1:1) and higher (3:1) alcohol: acid proportions (Figure 3). The esterification degrees of the four fatty acids were similar after 8-h with (2:1) or (3:1) excess of 1-butanol, but the reaction proceeded at a lower rate in the last case. The use of a stoichiometric proportion of the substrates afforded lower esterification yields and reaction rates than those observed in the standard assay with a 2:1 proportion. 

According to these results, the presence of a moderate excess of the alcohol produced the highest esterification yields in the shortest times with all the fatty acids used as acyl donors. Therefore, the following experiments were performed setting the molar ratio of acceptor and donor at 2:1. 

#### 2.4.2. Substrates Concentration

**Figure 4:**
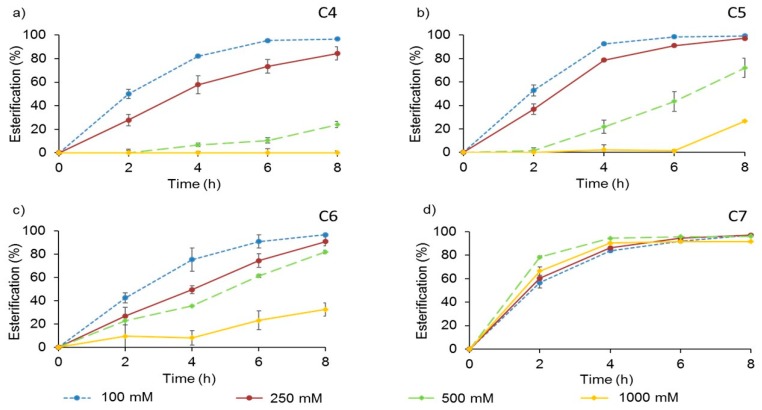
Effect of the substrates’ concentration in the esterification, catalyzed by AMNP-GA-OPEr, of 1-butanol and each VFA: (**a**) butanoic (C4); (**b**) pentanoic (C5); (**c**) hexanoic (C6); and (**d**) heptanoic (C7) acid. Reactions were performed in isooctane, at 25 °C and 100 rpm with an alcohol: acid 2:1 molar ratio and 11 U of the catalyst.

Maintaining the 2:1 molar ratio for the two substrates, their concentrations were accordingly changed between 100–1000 mM for each VFA tested and up to 2 M for 1-butanol. The rest of the parameters of the reaction catalyzed by AMNP-GA-OPEr remained unchanged. The data presented in Figure 4 indicate that at initial 1-butanol:VFA concentrations up to 500 mM:250 mM, the reaction yields surpassed 80% at the final reaction time (8 h). 

However, the highest concentrations negatively influenced the esterification degree, except for the heptanoic acid. In this case, the reaction rates and esterification degrees reached with all the assayed concentrations had comparable patterns, with more than 80% conversion upon 4 h of reaction. 

The optimal reaction times and concentration ranges for each one of the substrates tested can be deduced from these experiments. For the synthesis of butyl butyrate, a VFA concentration of 250 mM and a reaction time of 8 h could be used. For the valeric acid ester the reaction time would be reduced to 6 h for that same concentration and in the case of the hexanoic acid ester the most appropriate conditions would be 8 h of reaction and a VFA concentration of 500 mM. Finally, around 0.9 M of butyl heptanoate can be produced with this catalyst in 4 h.

#### 2.4.3. Branching of the Acyl Donor

In addition to the straight-chain VFA we also assayed two isoacids commercially available, the isobutyric (2-methylpropanoic acid) and isovaleric acids (3-methylbutanoic acid), as substrates of OPEr. These branched molecules have four and five-carbon atoms, as the butyric and valeric acids, respectively, and can serve to describe if ramifications affect the activity of AMNP-GA-OPEr. The synthesis of the isobutyric ester passed more slowly than that of the butyric ester, although the final yield after 8 h was very similar in both cases. However, the synthesis of the isovaleric ester was extremely inefficient, with maximum reaction yields lower to 20% (data not shown). The different position of the methyl branch in both substrates, summed to the higher chain length of the C5 isoacid could impede its access to the active center of the lipase.

### 2.5. Operational Stability of AMNP-GA-OPEr 

The stability of the nanobiocatalyst was verified in successive cycles of esterification. To facilitate the comparison of the results, we selected an acid concentration of 250 mM, the highest concentration with which the esterification yields surpassed 80% in 8-h reactions for all the acids tested. 

**Figure 5:**
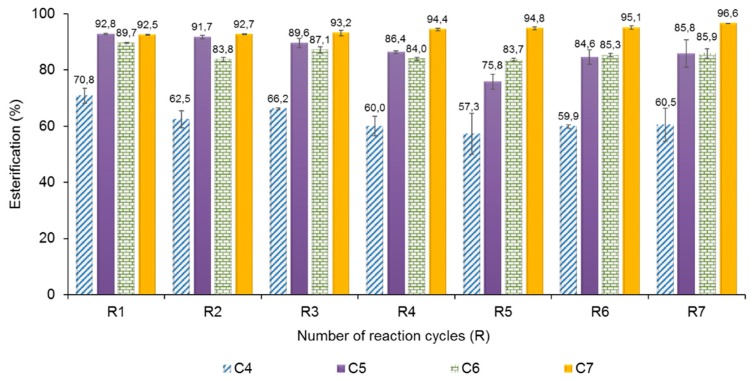
Operational stability of the catalyst AMNP-GA-OPEr, across seven cycles of esterification with 500 mM of 1-butanol and 250 mM of each VFA. Reactions were performed in isooctane, at 25 °C and 100 rpm with 11 U of the catalyst added in the first reaction cycle.

Figure 5 shows the esterification percentage achieved for each acid, across 7 consecutive cycles of reaction. The bar plots demonstrate few (<10%) or none activity loss throughout the successive reaction batches. As expected from the results from of the previous assays, the maximum loss was observed for reactions with butyric acid, and it was null for the synthesis of the heptanoic ester.

### 2.6. Characterization of the Nanoparticles and Nanobiocatalysts

**Figure 6:**
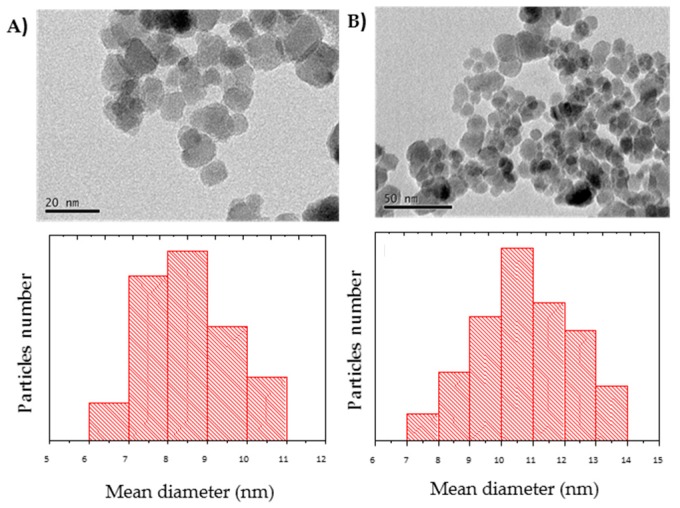
TEM micrographs and size distribution of: (**A**) nude magnetic nanoparticles (MNPs) and (**B**) amino-functionalized nanoparticles (AMNPs).

According to the information provided by the manufacturer, the SiMAG-Octyl particles have a hydrodynamic diameter of 1 µm and contain maghemite covered by a non-porous silica shell that harbors the functional octyl groups for hydrophobic interaction. The nanoparticles of magnetite used in our laboratory were visualized by Transmission Electron Microscopy (TEM) before and after amino-functionalization (Figure 6A,B), showing a homogeneous and spherical morphology, typical of Fe_3_O_4_ phases [36,37]. Their respective size distribution indicated an average particle diameter between 6-14 nm. As observed in the plot, the size distribution became larger for the coated sample. 

**Figure 7:**
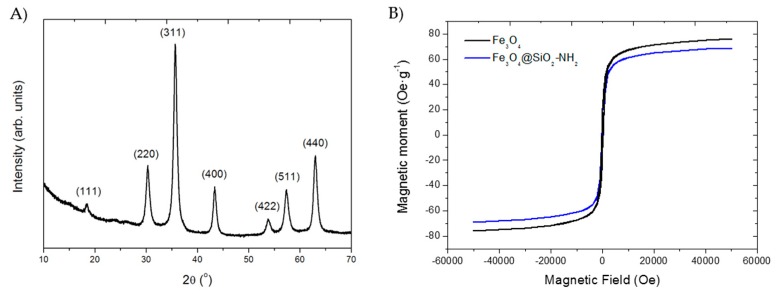
(**A**) XRD pattern of nude MNPs. The hkl Miller indexes corresponding to the six diffraction maxima are indicated on the top of each signal. (**B**) Variation of the magnetic moment *vs.* applied field. Black line is MNPs and blue line is AMNPs.

The XRD pattern of nude MNPs (Figure 7A) showed six maxima at around 18.3°, 30.3°, 35.7°, 43.3°, 53.7°, 63.1°2θ values. All diffraction maxima can be indexed to a cubic symmetry with space group Fd3m (JCPDS database No 82-1533), compatible with a spinel-type structure characteristic of Fe_3_O_4_ phase. The corresponding hkl Miller indexes are also shown in the Figure 7A. The estimation of the average crystallite size of the sample was carried out using the Scherrer formula [38] θ_hkl_ = 0.89λ/βcosθ where θ_hkl_ is the calculated average crystallite size, 0.89 is, assuming spherical particles, the shape factor, θ is the Bragg’s angle, β is the full-width at half-maximum (FWHM) of the experimental diffraction maxima and λ the X-ray wavelength. The average crystallite size of nude MNPs was estimated to be 15 nm. In addition, in order to perform a better structural characterization, the XRD pattern was analyzed by Rietveld method. The results suggest that both magnetite and maghemite are present in the sample [39].

The hysteresis cycles obtained for the MNPs and AMNPs samples showed a strong magnetic response to the application of a variable magnetic field (Figure 7B). The saturation magnetization (Ms) of the MNPs was 76 emu/g, while for the AMNPs this value was smaller (69 emu/g) probably due to the coating layer of APTS [36,37,40]. The amount of amino groups grafted to the surface of the nanoparticles was determined to be 10.5 µmols/g of carrier.

**Figure 8:**
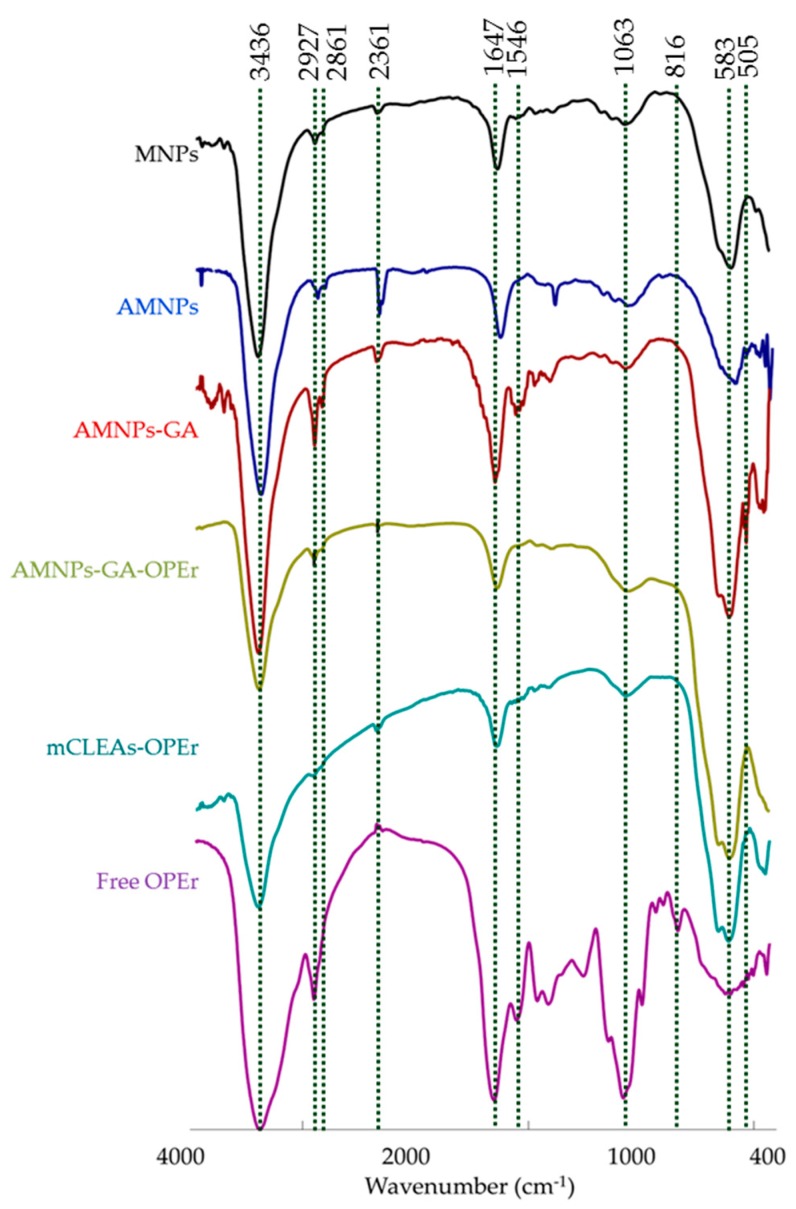
FTIR spectra of bare magnetite nanoparticles, aminated carriers, covalently-linked nanobiocatalys and free lipase OPEr.

FTIR spectra of the aminated carriers (activated with GA or not) and the two covalent nanobiocatalysts (Figure 8) displayed an absorption band with maximal intensity centered around 580 cm^−1^, that can be attributed to the Fe-O in the inverse spinel-type structure of the Fe in tetrahedral positions [41]. The band around 1060 cm^−1^ observed in the spectrum of the functionalized nanoparticles corresponded to the presence of SiOH and Si-O-Si groups [40,42] and that observed at approximately 800 cm^−1^ can be assigned to the bending vibration mode of the Si-O bonds [43]. The band centered at 1623 cm^−1^ in the spectrum of the AMNPs indicated the presence of the N-H stretch vibrations from the amino groups. The bands around 2927 and 2860 cm^−1^ correspond to –C-H stretching vibrations. These signals are more intense in the spectrum of the AMNP-GA carrier indicating the incorporation of GA (as monomer or as polymeric forms), either as monomers or as polymers, to the surface of the carrier. The widening of the peak centered at 1647 cm^−1^ in the spectrum of the AMNP-GA carrier might be due to activation with glutaraldehyde, as a multiplicity of small signals is observed in the C=O region (1730–1665 cm^−1^). The spectrum of the free protein shows the amide I and II bands around 1647 and 1546 cm^−1^ characteristic of the to the peptide linkage, and they are also observed in the spectra of the two covalent nanobiocatalysts.

**Figure 9:**
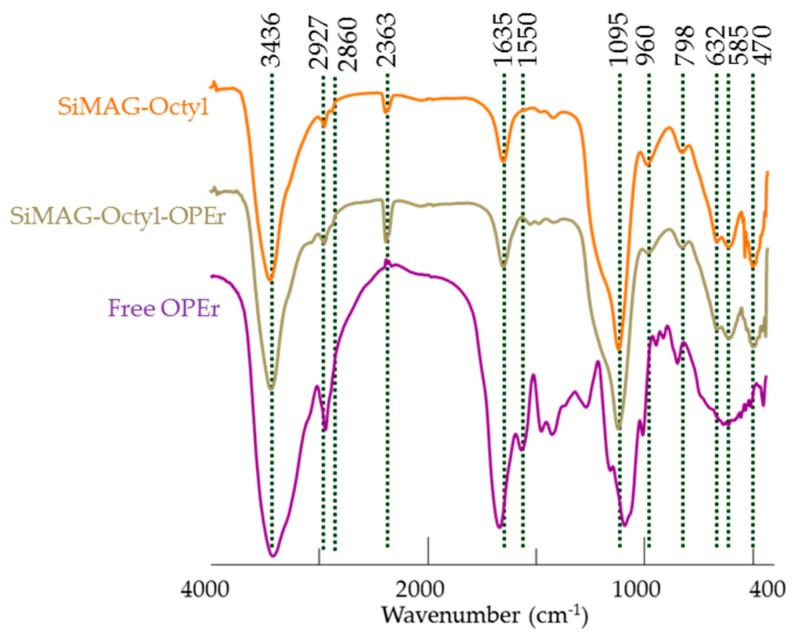
FTIR spectra of commercial SiMAG-Octyl carrier, OPEr immobilized to the carrier by hydrophobic attachment and free lipase OPEr.

On the other hand, the spectra of the commercial SiMAG-Octyl carrier and the nanobiocatalyst immobilized by hydrophobicity (Figure 9) display also the bands corresponding to the presence of magnetite and a silan coating (585, 798, 960, 1095 cm^−1^). In these samples, the absorption band around 1095 cm^−1^ is very intense probably because of a thick silica coating. The presence of the protein in SiMAG-Octyl-OPEr is confirmed by the weak band at 1550 cm^−1^.

## 3. Discussion

### 3.1. Synthesis of the Magnetic Nanobiocatalysts with OPEr and Comparison of Their Activity in Hydrolysis of pNPB

The use of biocatalysts immobilized on magnetite carriers is a very attractive option. The nanoparticles remain in suspension while the enzymatic reaction takes place and, at the end, a magnetic field is used for their quick capture and recovery, allowing also an easy separation of the reaction products. In the current work, we have used three approaches for immobilization of crudes containing the versatile lipase OPEr on the surface of functionalized magnetic nanoparticles. For covalent attachment, nude commercial nanoparticles of magnetite were functionalized with amino-propyl groups. The value measured for the Ms of the commercial MNPs was found to be lower to the 92 emu/g expected for bulk magnetite [44], which is compatible with the presence of a maghemite phase (Fe_2_O_3_) that has lower magnetization than magnetite (82 emu/g) [45]. The Ms value of the AMNPs decreased to 69 emu/g due to the coating layer of APTS [36,37,40], which increases the amount of non-magnetic substance present in the sample, and therefore, leads to a reduction in general magnetization. The presence of the silica coating was confirmed by FTIR, as well as the incorporation surface amino groups, which was also measured in a colorimetric assay.

Before adding the protein solution, the AMNPs were activated with glutaraldehyde. Due to the reactivity of glutaraldehyde, the alternative of particle coupling one-to-one in the absence of the enzyme cannot be excluded, although this fact did not impede the attachment of the protein to the activated carrier. The interactions between the AMNP-GA carrier and the proteins have been described to rely in several mechanisms: ionic adsorption, hydrophobic interaction, and covalent binding. As these supports are heterofunctional, their interaction with enzymes depends mainly on the ionic strength of the immobilization buffer [46]. The method applied to prepare AMNP-GA-OPEr involved the use of 100 mM buffer pH 7, and an immobilization time of 24 h. These conditions lead to the formation of covalent bonds between the reactive aldehyde groups from GA and the nucleophiles of the protein surface [47]. Since immobilization was performed at neutral pH, the proteins would more probably react with the carrier through their most reactive amino groups, rather than through the ε-amino of lysine located in its surface, that have a quite alkaline pK value. The catalyst obtained by this procedure is expected to maintain OPEr in a rigid conformation, due to the possibility of multipoint attachment [48,49]. As the lipase was not purified, the other proteins in the crude would also interact with the aldehyde groups of GA and get linked to the carrier, hampering the immobilization of OPEr at those positions and justifying the considerably high residual activity detected in the supernatants (Table 1). The data reported for covalent immobilization of lipases on AMNP-GA show a big variability, with yields between 50-90% depending on the initial amount of enzyme or the glutaraldehyde concentration, and activity recoveries of 30-96% [50,51,52]. In the current work, the recovered activity for AMNP-GA-OPEr was 49% and the activity loss could be due to an inefficient orientation of some OPEr molecules that avoids the entry of substrates to the active site [49], or to inactivation by the long exposure to GA.

The second method assayed for covalent immobilization of OPEr consisted in preparing mCLEAs. To do so, we followed the procedure described by Kim et al. [12] that comprises the pre-activation of the AMNPs with GA, leaving the protein solution in 10 mM buffer pH 8 to interact with the activated carrier for 2 h before adding the precipitant (ammonium sulfate) and the crosslinker (GA). This slightly alkaline pH probably does not influence very much the charge of the amino groups of the side chains of Lys. However, the low ionic strength of the buffer and the short contact time between the proteins and the aldehyde groups may facilitate the creation of quick ionic interactions instead of covalent bonds [47]. Indeed, once the mCLEAS are made, all the proteins in the crude are covalently immobilized, but the first ionic interaction with the carrier would probably condition the geometry of the final catalyst. In this case, 97% of OPEr was immobilized, as the presence of other proteins in the crude was not a factor limiting its precipitation and crosslinking, and the recovered activity was 74%. In fact, some protocols describe the incorporation of additional proteins (co-feeders) when synthetizing CLEAs as a tool to get preparations with enhanced activity [53]. Some lipases have been successfully immobilized as mCLEAS by this or other similar procedures and tested in reactions of hydrolysis [12] and synthesis [14,52].

On the other hand, the enzyme was also immobilized by non-covalent deposition onto SiMAG-Octyl, a hydrophobic magnetic support functionalized with octyl groups. This same functionality is used in the chromatography columns used for purification of OPEr from enzyme crudes, as the protein is extremely hydrophobic [23] and, as expected, 99% of the activity remained attached to the carrier. Hence, various positive effects can be achieved by means of the hydrophobic immobilization of OPEr. On one side, the final catalyst contains nearly pure OPEr, as it is the most hydrophobic molecule in the crudes, while the bulk of proteins that are not hydrophobic, are washed away. Keeping in mind that we worked with an enzyme crude, this fact marks an outstanding difference with the two covalent preparations of OPEr. Besides, the alkyl groups located on the surface of the carrier could create strong interactions with the internal surface of the lid or the area around the active centre, stabilizing the open form of OPEr which, in other lipases, has been described to cause hyper-activation [9,54,55,56]. However, it does not seem to be true for OPEr, given that the recovered *p*NPB activity for SiMAG-Octyl-OPEr amounted to 64% in spite of the total immobilization of the lipase. Thus, we have not observed the phenomenon of interfacial activation described for many lipases, and this activity loss in the immobilized catalyst could be due to protein crowding and inaccessibility to the catalytic site. The reports on immobilization of lipases on MNPs with octyl groups are scarce, but many works deal with their hydrophobic attachment to octyl agarose beads with yields around 100% and activity recoveries over 100%, as explained before [55,57,58]. 

The three nanobiocatalysts showed to be active, easily recoverable from the reaction medium in the presence of a magnetic field, and had long-term stability far superior to those described for other immobilized lipases [59,60]. As a last step of the characterization of their behavior in hydrolysis of *p*NPB, we determined their specific activity. Despite the excellent immobilization yields of the non-covalent SiMAG-Octyl-OPEr, its specific activity in this reaction was comparable to that of the AMNP-GA-OPEr, and both were around 1.5-times lower than for mCLEAs-OPEr (Table 1). The differences in the specific activity of the two covalent preparations may be due to the higher enzyme load immobilized as mCLEAs. The specific activity of the hydrophobic carrier was lower than expected from the lack of residual activity after immobilization, which suggests that a part of the OPEr molecules could have been buried during enzyme’s deposition or have their active centers hidden and then unavailable. If so, the specific activity could be improved adjusting the optimal amount of lipase offered for immobilization. 

### 3.2. The OPEr Magnetic Nanobiocatalysts Catalyze the Synthesis of the Butyl Esters of Straight-Chain Fatty Acids C4-C7 with Different Efficiency

Some VFA esters, and among them their butyl derivatives, are widely used as flavors and fragrances conferring different sensory characteristics depending on the length of their acyl chain. These compounds were chosen as a simple, but biotechnologically interesting model, to evaluate if OPEr maintained its synthetic activity after immobilization. These substrates also allowed testing the effect of their chain-length on the catalytic efficiency of the three OPEr preparations, which is probably influenced by the way in which the lipase was immobilized. All the OPEr-nanobiocatalysts were able of promoting the synthesis of the esters at more or less similar levels as the free enzyme, with yields that generally ranged between 75% and 100% at the final reaction time. However, the chain length of the acids exerted different effects on the time-course of the esterification. As observed in Figure 2, AMNP-GA-OPEr showed, in most cases, superior efficiency to SiMAG-Octyl-OPEr and mCLEAs-OPEr, converting nearly 100% of each limiting substrate into its corresponding ester in 4 h. Compared with the data registered for the free OPEr, the reaction rates observed with this preparation were better if butyric acid was the substrate, very similar for valeric and hexanoic acids, and lower for heptanoic acid. With this particular substrate, the yield after 4-h esterification was around 20% lower than the measured for the free enzyme and SiMAG-Octyl-OPEr, but at the end of the reaction the yields equaled except for the mCLEAs-OPEr. When the catalyst was immobilized by hydrophobic adsorption, the reaction rate increased proportionally with the VFA length. This agrees with data published for the commercial lipases Novozym 435, Lipozyme-TLL-IM, and Lipozyme-RM-IM, that are immobilized by adsorption [30]. 

The synthesis reactions catalyzed by lipases are usually explained by either Ping-Pong Bi-Bi or ternary complex (order-Bi-Bi) mechanisms, in which the first step is the binding of the acyl donor to form an acyl-enzyme intermediate [25]. So, the different efficiencies observed for the catalysts would depend mainly on their affinity and stability against each of the fatty acids. Lipases display less affinity for small-chain substrates because they can inhibit their catalytic action [61]. In spite of the fact that the reactions with the nanobiocatalysts were conducted in an organic medium, without added water, the acids’ dissociation can induce a local decrease in the pH of the water layer of the enzyme [62]. Pires-Cabral et al. [63] reported that the acid concentration in the micro-aqueous environment of the immobilized lipase of *C. rugosa* was superior to that existing in the bulk medium. Thus, a plausible reason to explain the above results would be that in AMNP-GA-OPEr the enzyme is fixed in a rigid conformation and thus has better stability to acidic substrates than the free enzyme. On the contrary, the enzymatic activity of SiMAG-Octyl-OPEr, immobilized by simple adsorption and hence with a certain degree of structural freedom, could be more susceptible to the inhibitory effect of the smaller VFA, which are more acidic and hydrophilic as their chain length decreases (C4 > C5 > C6 > C7). This effect would be less pronounced in reactions with free OPEr because the enzyme was added in solution providing 7% water, which decreases the acidity. Concerning mCLEAs-OPEr, they are probably the most rigid enzyme preparation of the three assayed, and the time course of the reactions with butyric, valeric and hexanoic acids were fairly similar. However, with the C7 substrate the esterification yields were 25% lower than those obtained with any of the other catalysts included in this study. Thus, the rigidity of the nanobiocatalysts seems to be play against when the substrates are larger.

These results show the different catalytic efficiency and stability of the immobilized preparations of OPEr and highlight the relevance of deepening on the advantages of testing several immobilization strategies for a given enzyme and application [5]. In the particular case of this versatile lipase, the synthesis of the different butyl esters with the covalent AMNP-GA-OPEr equaled or surpassed that of the other preparations.

### 3.3. Effect of Other Variables in the Enzymatic Synthesis of Butyl Esters of VFA Catalyzed by AMNP-GA-OPEr

After catalysis, the corresponding ester and water are released in equimolar amounts as the reaction products. Given that the reaction catalyzed by lipases is reversible, accumulation of the water produced in the medium could cause reversion to hydrolysis [21], and the presence of a molar excess of one of the substrates may help in deviating the balance towards the synthesis. Alcohol excess would be expected to be positive for the reaction because it contributes to neutralize the acidity of the other substrate of this reaction. However, large excesses of the alcohol can affect the water layer of the catalyst and then the esterification [26]. On the other hand, if the reaction yields are good enough, the use of stoichiometric amounts of the substrates is preferred for being cheaper, as it avoids the need to separate the unreacted substrate from the reaction product.

Hence, the proportion of the substrates in the reaction mixture can be a relevant parameter in synthesis reactions, and its impact on the enzymatic conversion should be analyzed. Here we found that two-fold excess of butanol provided the best results with all the fatty acids used as acyl donors. The influence of the molar proportion of substrates in similar reactions, catalyzed for different immobilized lipases, is uneven. Friedrich et al. [64] compared two preparations of the lipase B from *C. rugosa* in the synthesis of ethyl butyrate, finding that the molar ratio of the substrates had opposite effects on both preparations. Similarly, Martins et al. [65] tested two immobilized preparations of the lipase from *T. lanuginosus* in the synthesis of butyl butyrate, describing that the optimal proportions of the substrates were also different for each one of them. A later work of the same group reported that, in the optimal conditions for the commercial immobilized lipases Novozym 435, Lipozyme-TLL-IM and Lipozyme-RM-IM, the best yields were obtained using 3:1, 2.4:1, and 1.7:1 molar ratios, respectively. 

The influence of the substrates’ concentration was also analyzed, maintaining the 2:1 alcohol to acid molar proportion, and several conclusions can be drawn from the data. The excellent results obtained for the production of butyl heptanoate at the highest initial concentrations assayed, 1 M of heptanoic acid and 2 M of 1-butanol, virtually discard a negative effect of the alcohol on the activity of the catalyst. As already discussed, the focus should be placed on the acidic substrate, and in the high probability of these compounds affecting the local pH in the water layer of the enzyme, causing its inhibition or inactivation [62,63]. The results (Figure 4) suggest that the adverse consequences of using high concentrations of butyric, valeric, and hexanoic acids are less pronounced as the chain length of the volatile fatty acid used as substrate increases. 

The optimal concentration ranges of the different substrates for this reaction can be deduced from these experiments. For the synthesis of butyl butyrate, a VFA concentration of 250 mM and a reaction time of 8 h could be used. For the valeric acid butyl ester the reaction time would be reduced to 6 h for that same concentration and in the case of the hexanoic acid ester the most appropriate conditions would be 8 h of reaction and a VFA concentration of 500 mM. Finally, as already mentioned, the production of butyl heptanoate was the most efficient with this catalyst, even at shorter reaction times. 

The last step of this study addressed the assessment of the operational stability of AMNP-GA-OPEr. The decrease in the activity of a biocatalyst after repeated reaction cycles can be due to several factors, like enzyme leakage, substrate or product inhibition, or enzyme inactivation. No considerable activity loss was observed after seven reaction cycles, confirming the efficient recovery of the magnetic nanobiocatalyst and its good stability in all the instances, although it increased with the length of the chain of the VFA. 

## 4. Materials and Methods

### 4.1. Chemicals and Reagents

Butyric acid, valeric acid, isobutyric acid, isovaleric acid, hexanoic acid, heptanoic acid, 1-butanol, and *p*-nitrophenyl butyrate were purchased from Sigma-Aldrich (St. Louis, MO, USA). Other chemicals and solvents were of the purest available grade, provided by Sigma-Aldrich (St. Louis, MO, USA) or Merck (Darmstadt, Germany).

### 4.2. Strains, Culture Conditions, and Preparation of Enzyme Crudes

*P. pastoris* GS115 strain containing the *ope* gene was maintained and cultivated to produce OPEr as previously reported [20]. Cultures were then centrifuged (13,000 rpm, 4 °C) and fungal biomass discarded. Supernatants were concentrated by ultrafiltration in an YM3 Amicon device (Merck Millipore, Darmstadt, Germany) with a 50-kDa membrane. The crudes obtained were used without further purification.

### 4.3. Evaluation of Enzyme Activity and Protein Content

The standard assay to determine the activity of the catalyst was carried out monitoring at 410 nm the release of *p*-nitrophenol from hydrolysis of 1.5 mM *p*-nitrophenyl butyrate (*p*NPB) in 20 mM Tris-HCl pH 7.0 at room temperature, using a Shimadzu UV-160A spectrophotometer. One unit of activity (1 U) is defined as the amount of enzyme used to release 1 µmol of *p*-nitrophenol (ε_410_ = 15,200 M^−1^ cm^−1^) per minute under the defined conditions [66]. Protein concentration was determined by the BCA assay, using bovine serum albumin as standard, and by measuring the absorbance at 280 nm in a Nanodrop (NanoDrop 2000, Thermo Scientific, city, state abbreviation if USA, country).

### 4.4. Functionalization of Nude Magnetic Nanoparticles

Magnetic nanoparticles from Iolitec GmbH (Heilbronn, Germany) were functionalized with NH_2_ groups on their surface by treatment with (3-aminopropyl)triethoxysilane 99% (APTS, Sigma-Aldrich, city, state abbreviation if USA, country). MNPs (1 g, dry weight) were incubated with 10 mL of 130 mM APTS in methanol [67] and mixed at 80 rpm and 28 °C. After 16 h, they were washed three times with ethanol 50% and sonicated in an ultrasonic bath (Selecta, Spain) between washes. Finally, the AMNPs were dried at 65 °C in an aeration oven.

### 4.5. Characterization of the Nanoparticles

The amount of amino groups bound to the magnetic support was calculated according to the procedure described by del Campo et al. [68]. Morphological analysis was carried out by transmission electron microscopy (TEM) using a JEM HITACHI S-4800. Fourier Transform infrared (FTIR) spectra were collected using a FT/IR-4200 FTIR spectrometer (Jasco, Tokyo, Japan) in the spectral range 4000–400 cm^−1^, with a spectral resolution of 4 cm^−1^ in transmittance mode. The samples were analyzed as KBr pellets. XRD patterns were recorded using a Siemens D5000 diffractometer equipped with a Cu anode (Cu K_α_ radiation) and a LiF monochromator to study the structural and phase analysis. The Rietveld refinement of XRD patterns was performed using the TOPAS v4.2 software (Bruker AXS, Karlsruhe, Germany) and taking into account the crystallographic information for the different phases from Pearson’s crystal structure database for inorganic compounds [69]. The average crystallite size of nude MNPs was also estimated by X-ray pattern using the Debye-Scherrer formula [70]: D_hkl_ = 0.89·λ/β·cosθ where D_hkl_ is the average crystallite size, 0.89 is the shape factor (assuming spherical particles), λ is the X-ray wavelength used (1.5406 Å for Cu K_α_), β is the full-width at half-maximum (FWHM) of the experimental diffractions and θ is the Bragg’s angle. The magnetic measurements were performed on a Quantum Design XL-SQUID magnetometer (Quantum Design International, San Diego, CA, USA). Hysteresis measurements were taken with applied field range from 0 to 50,000 Oe (5T).

### 4.6. Immobilization of Crudes with the Recombinant Versatile Lipase from O. piceae

The crudes with OPEr were immobilized by three procedures. In all cases, the immobilization yield (%) was calculated from the difference between offered activity and residual activity in the supernatant at the end of the immobilization period. The activity recovery was determined by considering the OPEr activity initially offered for immobilization and the activity of the immobilized catalyst.

#### 4.6.1. Covalent Immobilization on Amino-Functionalized Magnetic Nanoparticles Activated with Glutaraldehyde 

First, AMNPs (1 g dry weight) were activated by incubation with 40 mL of 250 mM glutaraldehyde (Sigma-Aldrich) in water, for 3 h. Then, AMNPs modified with glutaraldehyde (AMNPs-GA), with surface aldehyde groups, were washed six times with water to remove the residual glutaraldehyde and one more time with 20 mM Tris-HCl buffer solution, pH 7.

AMNPs-GA (1 g) were incubated with OPEr crudes (0.07 mg protein/mg AMNP-GA) in a final volume of 30 mL (100 mM Tris-HCl buffer solution, pH 7) for 24 h at 28 °C with rotational mixing at 80 rpm (Multi Bio RS-24, Biosan). After immobilization, AMNP-GA-OPEr were thoroughly washed with 20 mM Tris-HCl buffer solution, pH 7 to remove the unbound proteins, and maintained in the same buffer at 4 °C until used. 

#### 4.6.2. Immobilization as Magnetic CLEAS

*mCLEAs* were prepared as reported by Kim et al. [12]. In brief, 1 g of AMNPs was allowed to react with 0.5% GA in Tris-HCl 10 mM pH 8 for 3 h at 25 °C and 200 rpm. After three washes, the crude protein solution (0.05 mg protein/mg carrier) was added to the GA-activated AMNPs in a final volume of 10 mL of the buffer and maintained for 2 h at 25 °C and 50 rpm. Then, 10 mL of ammonium sulfate 4 M and 10 mL of the 0.5% GA solution were incorporated to the mixture and stirred at 250 rpm for 1 h at 25 °C and then at 4 °C for 24 h. The mCLEAs-OPEr were washed three times with the above buffer and four more with Tris-HCl 20 mM pH 7, and maintained in this buffer at 4 °C until used.

#### 4.6.3. Immobilization by Adsorption on Commercial Magnetic Nanoparticles Functionalized with Hydrophobic Octyl Groups

A commercial solution containing 250 mg of SiMAG-Octyl (Chemicell, Germany) was carefully mixed for 2 min at 28 °C with OPEr crudes containing 0.07 mg protein per mg of carrier in 5 mL 100 mM Tris-HCl buffer pH 7. The biocatalyst was stored in 20 mM Tris-HCl buffer solution, pH 7 at 4 °C.

### 4.7. Activity of Immobilized Enzymes

The hydrolytic activity of immobilized enzymes was measured by the hydrolysis of *p*-nitrophenyl butyrate as described in Section 4.3. The amount of biocatalyst used was 0.25 mg. The results were expressed as mU per mg of biocatalyst.

### 4.8. Esterification of Volatile Fatty Acids in Isooctane

A suspension containing 11 U of the immobilized biocatalyst (AMNP-GA-OPEr, mCLEAs-OPEr or SiMAG-Octyl-OPEr) in 20 mM Tris-HCl buffer pH 7 was added to a clean vial. A magnet was used to attract the magnetic catalyst in order to remove the buffer. The reaction mixtures (500 µL) contained the substrates in a molar ratio 2:1 (alcohol:acid) in isooctane, at a concentration of 100 mM of each one of the acids (C4-C7 straight-chain volatile fatty acids), and 2% hexane as internal standard. Each mixture was deposited in a tube containing the catalyst to start the reactions. The reactions with the free (soluble) enzyme were performed using the same conditions except for the presence of 7% water in the medium. The standard experiments were performed under the conditions detailed above, with rotational mixing at 100 rpm (Multi Bio RS-24, Biosan), at 25 °C for 8 h. For studying their operational stability, the biocatalysts were washed with 1 mL of isooctane and 1 mL of 20 mM Tris-HCl buffer pH 7 and used in a new reaction cycle under the same conditions. 

Some of the reaction parameters were individually modified to study their influence on the esterification yields and reaction rate. To test the effect of the molar ratio of the substrates, stoichiometric (1:1) and 3:1 molar proportions (alcohol:acid) were assayed. The influence of the acids’ concentration was analyzed in the range of 100 to 1000 mM. The esterification of two branched-chain volatile fatty acids, isobutyric acid and isovaleric acid, was also assessed under the standard conditions in reactions catalyzed by AMNP-GA-OPEr.

All these experiments were performed in duplicate, taking samples of 25 µL at 0, 2, 4, 6, and 8 hours to monitor the time-course of the reaction by gas chromatography/mass spectrometry, as described in Section 4.9. 

### 4.9. Monitoring Reactions by Gas Chromatography

As explained before, hexane was added to reactions as internal standard for gas chromatography/mass spectrometry analysis. The aliquots withdrawn from the reaction mixture, were deposited into a clean vial, and mixed with 25 µL of BSTFA (Sigma-Aldrich) for derivatization at 60 °C for 10 min. 1 µL of this solution was injected in a 7890A gas chromatograph coupled to a quadrupolar mass detector 5975C (Agilent, Palo Alto, CA). The injector and flame ionization detector were set up at 275 °C, and He (13 psi) was used as the carrier gas. The separation was carried out using a fused-silica capillary column DB5-HT (30 m × 250 µm × 0.1 µm, Agilent, Palo Alto, CA, USA). For analysis of volatile fatty acid esters, the oven was maintained at 80 °C for 1 min and then a temperature program was applied, with a 70 °C /min ramp rate to reach a final temperature of 240 °C. The peaks of substrates and products were identified from their retention time, compared to that of commercial standards. The esterification yields were calculated from a calibration curve of each product.

## 5. Conclusions

The versatile lipase OPEr has been immobilized on magnetic nanoparticles either by hydrophobic interaction, or by covalent attachment as mCLEAs or AMNPs-GA. All preparations were active in hydrolysis of *p*NPB and catalyzed the synthesis of butyl esters of VFA in very mild conditions. However, the reaction progressed at different rates, and in general terms AMNP-GA-OPEr seemed to be the most efficient and stable in the presence of acidic substrates. With this catalyst, the use of a moderate excess of the acids (2:1) had a positive effect on the reaction, and its reusability was good, maintaining esterification yields of 80–96% after seven successive cycles. In summary, this study reveals the excellent properties of the immobilized OPEr, its easy recovery and recycling, and its potential to catalyze the synthesis of chemicals of industrial interest in a green process.

## Figures and Tables

**Table 1:** Immobilization of OPEr onto magnetic nanoparticles. Lipase activity was determined using *p*NPB as the substrate. The amount of OPEr attached to the carriers was calculated subtracting the activity of the free enzyme in the supernatants before and after immobilization. The terms offered protein and offered activity refer to the amount of protein and lipase activity of the protein solution before immobilization.

Biocatalyst	Immobilization Type	Offered Protein (mg/mg Carrier)	Offered Activity (mU/mg Carrier)	Yield (%)	Specific Activity (mU/mg Carrier)
**SiMAG-Octyl-OPEr**	Hydrophobicity	0.07	1200	99	430 ± 60
**AMNP-GA-OPEr**	Covalent	0.07	1200	53	440 ± 20
**mCLEAS-OPEr**	Covalent	0.05	990	97	728 ± 2
